# Cryptanalysis and Improvement of "A Secure Password Authentication Mechanism for Seamless Handover in Proxy Mobile IPv6 Networks"

**DOI:** 10.1371/journal.pone.0142716

**Published:** 2015-11-18

**Authors:** Mojtaba Alizadeh, Mazdak Zamani, Sabariah Baharun, Azizah Abdul Manaf, Kouichi Sakurai, Hiroki Anada, Hassan Keshavarz, Shehzad Ashraf Chaudhry, Muhammad Khurram Khan

**Affiliations:** 1 Faculty of Information Science and Electrical Engineering, Kyushu University, Fukuoka, Japan; 2 Department of Computer Science, Kean University, Union, New Jersey, United States of America; 3 Malaysia-Japan International Institute of Technology, Universiti Teknologi Malaysia, Kuala Lumpur, Malaysia; 4 Advanced Informatics School, Universiti Teknologi Malaysia, Kuala Lumpur, Malaysia; 5 Institute of Systems, Information Technologies and Nanotechnologies (ISIT), Fukuoka, Japan; 6 Department of Computer Science and Software Engineering, International Islamic University, Islamabad, Pakistan; 7 Center of Excellence in Information Assurance, King Saud University, Riyadh, Saudi Arabia; University of South Australia, AUSTRALIA

## Abstract

Proxy Mobile IPv6 is a network-based localized mobility management protocol that supports mobility without mobile nodes’ participation in mobility signaling. The details of user authentication procedure are not specified in this standard, hence, many authentication schemes have been proposed for this standard. In 2013, Chuang et al., proposed an authentication method for PMIPv6, called SPAM. However, Chuang et al.’s Scheme protects the network against some security attacks, but it is still vulnerable to impersonation and password guessing attacks. In addition, we discuss other security drawbacks such as lack of revocation procedure in case of loss or stolen device, and anonymity issues of the Chuang et al.’s scheme. We further propose an enhanced authentication method to mitigate the security issues of SPAM method and evaluate our scheme using BAN logic.

## Introduction

Mobile devices have been experiencing rapid growth as people utilize these devices to access different types of services, including the Internet browsing, file sharing, video conferencing, and multimedia applications, anytime and anywhere [1]. This growth does not appear to halt any time soon even though mobile devices are faced with different challenges in using wireless technologies such as computation limitation, wireless communication bandwidth inadequacy, and security problems. The Mobile IPv6 (MIPv6) [2] is a standard of the Internet Engineering Task Force (IETF), that facilitates the roaming of the mobile nodes in the IPv6 network. This standardized protocol allows the mobile devices to roam inside the network by providing seamless connection to the network.

The nodes mobility must be transparent to the layers above the IP layer; the continuous connection can be seamless, and it may do not require any manual configurations. If the node has to connect to a different network connection during physical movement that utilizes a variant of the subnet prefix, then a mobile node (MN) is required to get a new IP address. If this does not take place, then the MN cannot be reached. In order for this seamless movement to take place, the Mobile IPv6 nodes utilize two addresses namely the Care-Of-Address (CoA) and the Home Address (HoA). The HoA is a permanent and static address, which can be utilized to connect to the MN despite the present location of the node, but the CoA is a dynamic and robust address, which changes according to the present location of the node. In order for the MN to be reached despite its location, the Mobile IPv6 establishes the HA (Home Agent) which functions as a proxy that is stationary [3].

The mobile IPv6 protocols are facing are several problems such as delay, packet loss, and signaling costs. Therefore, various mobility management protocols are suggested to increase the performance of the MIPv6, including, host-based such as the Hierarchical Mobile IPv6 (HMIPv6) [4], Fast Handover for Mobile IPv6 (FMIPv6) [5], and network-based such as the Proxy Mobile IPv6 (PMIPv6) [6]. Among these protocols, Proxy Mobile IPv6 (PMIPv6) gains fewer handover latency and signaling cost [7]. Proxy Mobile IPv6 (PMIPv6) is a network-based mobility management protocol, which offers mobility services for mobile nodes without the involvement of the mobile nodes in signaling communications. This particular protocol is being utilized as a variant of the wireless networks, including the 3GPP2, WiMAX, and the LAN networks as they need a low mobility signaling over the wireless links [8].

The Local Mobility Anchor (LMA), and the Mobile Access Gateway (MAG) are the main mobility entities in the PMIPv6 domain that provide seamless connectivity for the MN. The MAG typically runs on the access router, and manages mobility signaling instead of the MN. Subsequently, the MN in the PMIPv6 does not require any protocol stack modification in order to support the PMIPv6. The MAG and LMA manage the traffic transmitted to and from the MN using a bi-directional tunnel. Based on the MN view, the entire PMIPv6 domain appears as its home network [7].

Researchers have suggested various schemes of authentication for the PMIPv6 standard ever since it was first established in 2008, because the authentication procedure’s details are not specified in the RFC 5213 standard document. Chuang et al., [9] in 2013, suggested the authentication approach known as the SPAM. Nevertheless, the SPAM offers low packet loss and latency rates in comparison to many other schemes; however, it is prone to security threats such as impersonation and password guessing attacks. This study reveals that an attacker can act as a legitimate entity and attack when the mobile device is stolen or lost. In addition, this study demonstrates some present drawbacks in the scheme, including the lack of the revocation process and user anonymity problems. Moreover, the proposed improvement is suggested to make the SPAM secure against the security flaws mentioned above. Finally, the security and privacy of the proposed method is verified and discussed by utilizing the offered security theories and BAN logic, then authentication cost of the proposed method is compared with SPMA scheme.

The rest of this paper is organized in the following manner. The SPAM scheme is reviewed in Section 2. The cryptanalysis of the SPAM approach is established in Section 3. Section 4 provides our proposed solution. In Section 5, we assess the proposed approach by utilizing the security verification theorems. Finally, authentication cost of the proposed method is analyzed and compared to the SPAM scheme.

## Review of the SPAM Scheme

The SPAM includes three stages known as the initial registration, mutual authentication process for both the MAG and the MN, and the password changing process. The authentication credentials are stored in smart card under the assumption of using tamper-proof smart card. Table 1 describes the notations utilized in the SPAM scheme.

**Table 1:** Notations used in SPAM scheme.

Symbol	Description
*sv*	The AAA and LMA secret key
*ID*_*MN*_	MN identification
*ID*_*AAA*_	AAA identification
*ID*_*MAG*_	MAG identification
*PW*_*MN*_	Password of MN
*SK*_*i*−*j*_	Session key between entity (i) and entity, (j)
*E*_*SK*_*i*−*j*__(*M*)	Message M is encrypted using key SKi-j
*E*_*K*_(*M*)	Message M is encrypted using key K
*N*_*i*_	Nonce number i
*h( )*	One-way hash function
*PSK*	The symmetric key among the MAGs, the LMAs, and the AAA
∥	Concatenation
⊕	XOR operation

### Initial Registration

The mobile node receives certain credentials for further authentication during the initial registration with the authentication server, AAA. It is assumed that the communication channel between the MN and the AAA server is secure. The initial registration steps are as follows:

1. *MN* → *AAA*: The MN sends its ID and Password to the AAA server using secure channel.
2. The AAA server checks the ID and password on the MN and then computes the required values as follows. *c*_1_ = *h*(*ID*_*MN*_ ∥ *sv*), *c*_2_ = *h*(*PW*_*MN*_) ⊕ *c*_1_, *c*_3_ = *E*_*PSK*_(*ID*_*AAA*_ ∥ *sv*), *c*_4_ = *h*(*ID*_*AAA*_ ∥ *sv*), *c*_5_ = *h*(*sv*)
3. *AAA* → *MN*: The AAA stores *c*_1_, *c*_2_, *c*_3_, *c*_4_, *c*_5_, *h*(), *ID*_*MN*_ in the smart card and sends it to the MN.

**Fig 1:**
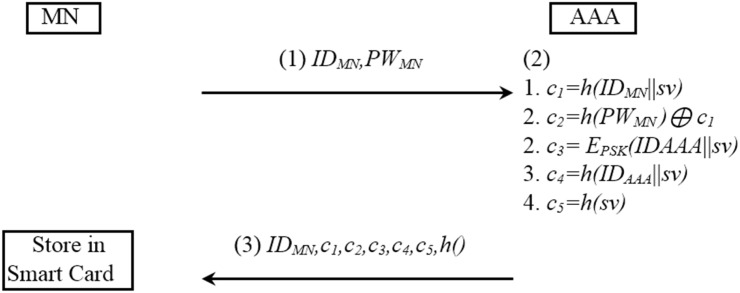
Initial registration procedure of SPAM method.

The initial procedure is described in Fig 1.

### Mutual Authentication between the MN and the MAG

There are two main sections in this mutual authentication; firstly, the MN’s authenticity is checked by the MAG prior to knowing its real ID, and secondly; the MN checks the MAG authentication. The mutual authentication between the MN, and the MAG is described in the following:

1. The user inserts a smart card and enters its ID and password. The smart card verifies whether the equation, *h*(*PW*_*MN*_) ⊕ *c*_2_ = *c*_1_, to check mobile user authentication. Then, it generates *N* and compute *AID*_*MN*_ = *ID*_*MN*_ ⊕ *h*(*c*_5_ ∥ *N*_1_) and *AUTH*_*MN*_ = *h*(*c*_1_ ∥ *N*_1_).
2. *MN* → *MAG*: The authentication request, *AID*_*MN*_, *c*_3_, *E*_*c*_4__(*AUTH*_*MN*_ ∥ *N*_1_), is generated by the MN and sent to the MAG.
3. The MN verification by the MAG: After receiving authentication request, the MAG decrypts *c*_3_ to obtain *ID*_*AAA*_ and *sv* using *PSK*, which is a pre-shared symmetric key. Then, the *AUTH*_*MN*_ and *N*_1_ are retrieved by decrypting *E*_*c*_4__(*AUTH*_*MN*_ ∥ *N*_1_) using *c*_3_. To obtain the *ID*_*MN*_, the MAG computes c5 and gets *ID*_*MN*_ = *AID*_*MN*_ ⊕ *h*(*c*_5_ ∥ *N*_1_). After computing *c*_1_ = *h*(*ID*_*MN*_ ∥ *sv*), the MAG can verify the MAG authentication by checking the value of *AUTH*_*MN*_ = *h*(*c*_1_ ∥ *N*_1_) to the value of *AUTH*_*MN*_ obtained from *E*_*c*_4__(*AUTH*_*MN*_ ∥ *N*_1_). If both *AUTH*_*MN*_ value are the same, the MN is authenticated and the MAG generates *N*_2_, *SK*_*MN* − *MAG*_ = *h*(*c*_1_ ∥ *N*_1_) that is a session key between the MAG and the MN, and *h*(*ID*_*MAG*_ ∥ *N*_2_).
4. *MAG* → *MN*: The MAG reply *ID*_*MAG*_, *E*_*c*_4__((*N*_1_ + 1)∥*N*_2_ ∥ *h*(*N*_2_ ∥ *ID*_*MAG*_)) back to the MN.
5. The MAG verification: The MN decrypts the *E*_*c*_4__((*N*_1_ + 1)∥*N*_2_ ∥ *h*(*N*_2_ ∥ *ID*_*MAG*_)) and obtains (*N*_1_ + 1) and *N*_2_. Then, it checks the value of *h*(*N*_2_ ∥ *ID*_*MAG*_) and (*N*_1_ + 1) for the MAG authentication. After verifying the MAG authenticity, the MN generates a session key, *SK*_*MN* − *MAG*_ = *h*(*N*_1_ ∥ *N*_2_).
6. *MN* → *MAG*: The MAG computes *E*_*SK*_*MN* − *MAG*__(*N*_2_ + 1), and sends it to the MAG.
7. The MAG decrypts the encrypted message using the session key and checks (*N*_2_ + 1) to prevent replay attack.

**Fig 2:**
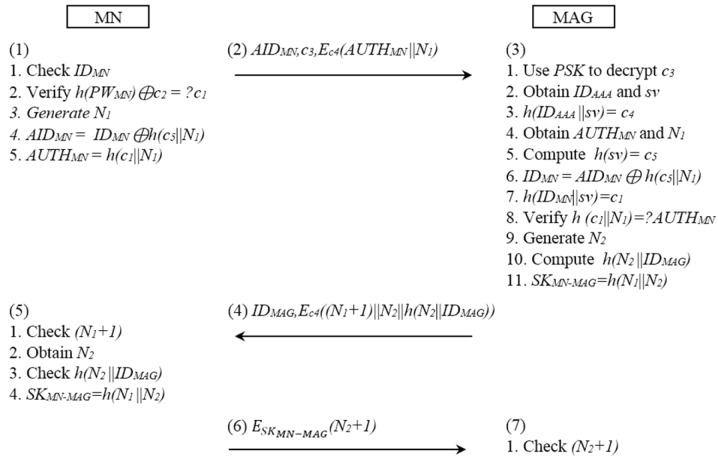
The SPAM authentication procedure between the MN and the MAG.

Fig 2 shows the communication between the MN and the MAG.

After mutual authentication between the MN and the MAG, the mutual authentication between the MAG and the LMA is processed in the SPAM method. The details of this authentication procedure are as follows.

1. The MAG generates *N*_3_ to compute *h*(*N*_3_ ∥ *ID*_*MAG*_).
2. *MAG* → *LMA*: The authentication message, *ID*_*MAG*_, *E*_*PSK*_(*N*_3_ ∥ *h*(*N*_3_ ∥ *IDMAG*) to the LMA.
3. The LMA decrypts the received message from the MAG using PSK and retrieves *h*(*N*_3_ ∥ *ID*_*MAG*_) and *N*_3_. The LMA computes *h*(*N*_3_ ∥ *ID*_*MAG*_) and compares to the received *h*(*N* 3 ∥ *IDMAG*) and *N*_3_. Then, it computes *h*(*N*_3_ ∥ *ID*_*MAG*_) and compares to the received *h*(*N*_3_ ∥ *ID*_*MAG*_) to check the MAG authenticity. Finally, it generates *N*_4_ and computes the session key, *SK*_*LMA* − *MAG*_ = *h*(*N*_3_ ∥ *N*_4_), if the MAG is authentic, otherwise, it drops the message.
4. *LMA* → *MAG*: The MAG replies *ID*_*MAG*_, *E*_*PSK*_((*N*_3_ + 1)∥*N*_4_ ∥ *h*(*ID*_*LMA*_ ∥ *N*_4_)) back to the MAG.
5. *The LMA verification:* The MAG decrypts *E*_*PSK*_((*N*_3_ + 1)∥*N*_4_ ∥ *h*(*ID*_*LMA*_ ∥ *N*_4_)) and obtains (*N*_3_ + 1) and *N*_4_. Then, it checks the value of *h*(*N*_4_ ∥ *ID*_*LMA*_) and (*N*_1_ + 1) for the MAG authentication. After verifying the MAG authenticity, the MAG generates a session key, *SK*_*LMA* − *MAG*_ = *h*(*N*_3_ ∥ *N*_4_).
6. *MAG* → *LMA*: The MAG computes *E*_*SK*_*LMA* − *MAG*__(*N*_4_ + 1), and sends it to the LMA.
7. The LMA decrypts the encrypted message using the session key and checks (*N*_4_ + 1) to prevent the replay attack.

**Fig 3:**
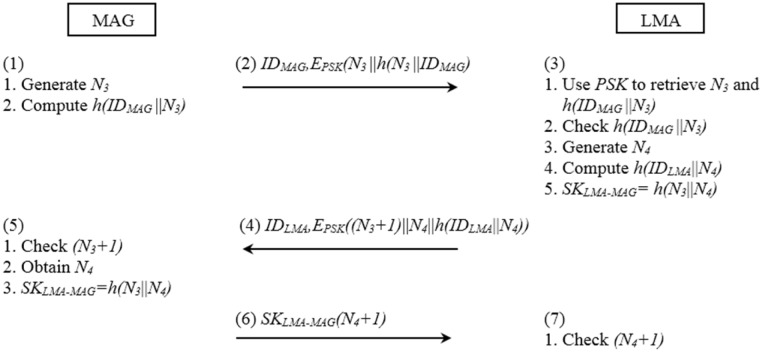
The authentication procedure between the MAG and the LMA.

The message exchange flow chart of mutual authentication between the LMA and the MAG is illustrated in Fig 3.

### SPAM Password Change Phase

The SPAM scheme provides the password change process. Mobile users are able to change their passwords without contacting other entities like the AAA server and the MAG. The procedure is described as follows:

1. The user inserts the smart card and enters his ID and password.
2. The smart card verifies user ID by checking *h*(*PW*_*MN*_) ⊕ *c*_2_ = *c*_1_. If the equation is correct, then lets user to enter new password, $PW_{MN}^{*}$. After receiving the new password, the smart card computes $c_{2}^{*}=c_{2}⊕h\left(PW_{MN}\right)⊕h\left(PW_{MN}^{*}\right)$ and replaces *c*_2_ by $c_{2}^{*}$.

**Fig 4:**
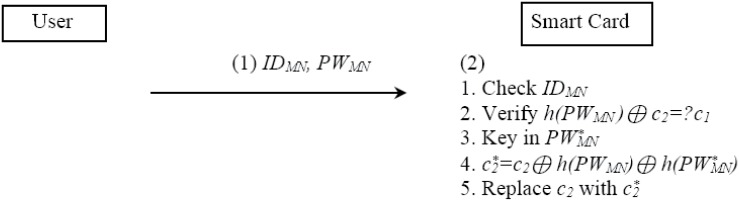
SPAM password change procedure.

The password change flow chart is described in Fig 4.

## Security Issues of the SPAM Method

This section discusses the security strengths of the authentication methods in the PMIPv6 using the assumption that smart cards are not exactly free from tampering. The suitable authentication method should fulfill some security and privacy criteria such as anonymity, mutual authentication, session key secrecy, and user unlinkability [10–15]. Furthermore, authentication schemes should secure enough against some security attacks such as session hijacking, denial of service, impersonation, replay, password guessing, man-in-the-middle, stolen-verifier, and eavesdropping attacks [16–24]. Therefore, we discuss the security and privacy of the SPAM method under the assumption that smart cards are not exactly free from tampering. In addition, the potential for utilizing smart cards in PMIPv6 that are tamper resistant are explained according to these researchers [25–31] by offering several examples. After that, the SPAM method’s security issues are discussed using certain evidences.

The conventional remote authentication using passwords [32, 33] utilizes a password table, which is stored in an authentication server. This kind of approach is susceptible to attacks on password, including password dictionary attacks, offline guessing attack, tampering of the password table, and corruption attacks. This also gives rise to an increase overhead for protecting and maintaining the password table. Therefore, many smart card based password authentication schemes that do not require a password table have been proposed [34–43] to improve security of the authentication protocols. However, these schemes remain vulnerable to sophisticated attacks that use offline password dictionary searches, observation of power consumption, or physically exposition of the chip to extract the data it stores [44].

Khan et al. [26] and Rhee et al. [29] claim that mobile devices, including smart phones, PDAs, and notebooks are not free from tampering and users’ data inside the mobile devices are susceptible to different forms of security attacks [31]. Various methods have been suggested to crack the security of smart cards in the past few years. For instance, Kocher et al. [45] proposed the potential of retrieving the smart card’s secret key by observing the smart card’s power consumption. The vulnerability of the smart card is observed through its power analysis attack [46]. Another form of the threat against the smart cards is the fault-based cryptanalysis, as demonstrated by Bellcore’s press release [47]. This attack occurs when an attacker initiates a particular form of fault into the mobile device and later retrieves the secrets embedded within according to the incorrect responses received from the mobile devices. Therefore, given the assumption of utilizing a non-tamper-proof smart card, many of the authentication methods in the PMIPv6 are susceptible to different forms of attacks like the impersonation attack; thus, making it is crucial to offer an appropriate method of authentication according to the assumption of the non-tamper-proof smart card.

This paper assumes that the attacker could have complete control of the channel of communication between the MAG and the MN, and he/she would be able to change, insert, and tap into any messages of communication. In the following sections, the security and privacy issues of the SPAM method are discussed.

### The MN Impersonation Attack

Mobile devices such as smartphones, PDAs, and Tablets are vulnerable to threats such as stolen or loss. In addition, most of the authentication mechanisms use smart card to store critical information such as secret keys, passwords, and encryption functions. Therefore, if an attacker access to smart card inside mobile devices and steal the keys, even if he leaves the mobile device intact, he can impersonate legitimate user or access point [26, 48](Khan and Kumari, 2014; Wei-Chi and Chang, 2005). In SPAM method, the information are stored in smart card, hence impersonation attack can be launched. The smart card in the SPAM method contains (*ID*_*MN*_, *C*_1_, *C*_2_, *C*_3_, *C*_4_, *C*_5_, *h*()), if an attacker accesses to this smart card secrets, and sniffs the first message, (*AID*_*MN*_, *c*_3_, *E*_*C*_4__(*AUTH*_*MN*_ ∥ *N*_1_)) between the MN and the MAG in login phase, he can impersonate the MN as follows:

1. First, an attacker generates his own nonce, $N_{1}^{*}$, then computes $AID_{MN}=ID_{MN}⊕h\left(C_{5}∥N_{1}^{*}\right)$, and $AUTH_{MN}=h(C_{1}∥N_{1}^{*}$ using retrieved secrets from smart card an login request message, *ID*_*MN*_, *C*_1_, and *C*_5_.
2. An attacker generates authentication request, $AID_{MN},C_{3},E_{C_{4}}\left(AUTH_{MN}∥N_{1}^{*}\right)$, and sends it to the MAG.
3. The MAG decrypts *C*_3_ using *PSK* and obtains *ID*_*AAA*_ and *sv*. Then, calculates *C*_4_ = *h*(*ID*_*AAA*_ ∥ *sv*) to decrypts *Ec*4(*AUTH*_*A*_||*N* ∗ 1) to obtain the value of *AUTH*_*A*_ and *N*_1*_. The MAG computes $ID_{MN}=AID_{A}⊕h\left(C_{5}∥N_{1}^{*}\right)$ and *h*(*ID*_*MN*_ ∥ *sv*) = *C*_1_. Finally, for checking MN authentication, the MAG compares the value of the $AUTH_{MN}=h\left(C_{1}∥N_{1}^{*}\right)$ to the value of *AUTH*_*MN*_ obtained from $Ec4(AUTH_{MN}∥N_{1}^{*})$. It is clear that the value, *AUTH*_*MN*_, which is retrieved from $\left(AUTH_{MN}∥N_{1}^{*}\right)$, is equal to the value, *AUTH*_*MN*_, retrieved from $AUTH_{MN}=h\left(C_{1}∥N_{1}^{*}\right)$, because *AUTH*_*MN*_, is generated using the values, *C*_1_, *C*_2_, and $N_{1}^{*}$, which can be captured or generated by an attacker. This means an attacker is authenticated to the MAG successfully.

### The MAG Impersonation Attack

Similar to the MN impersonation attack, we assume that an attacker retrieved the smart cart secrets, (*ID*_*MN*_, *C*_1_, *C*_2_, *C*_3_, *C*_4_, *C*_5_, *h*()), and sniffed the login request, (*AID*_*MN*_, *c*_3_, *E*_*C*_4__(*AUTH*_*MN*_ ∥ *N*_1_)). An attacker can impersonate the MAG as follows:

1. An attacker decrypts *E*_*C*_4__(*AUTH*_*MN*_ ∥ *N*_1_) to get *N*_1_, then generate $N_{2}^{*}$, and selects a fake $ID_{MAG}^{*}$. Finally, computes $(E_{C_{4}}\left(\left(N_{1}+1\right)∥N_{2}^{*}∥h\left(N_{2}^{*}∥ID_{MAG}^{*}\right),ID_{MAG}^{*}\right)$ and sends it back to the MN.
2. The MN decrypts $E_{C_{4}}(\left(N_{1}+1\right)∥N_{2}^{*}∥h\left(N_{2}^{*}∥ID_{MAG}^{*}\right)$ to obtain (*N*_1_ + 1) and $\left(N_{2}^{*}\right)$. Then, it checks the value, $h(ID_{MAG}^{*}∥\left(N_{2}^{*}\right))$, and (*N*_1_ + 1) for the MAG authentication. As the value, *N*_1_ is the original nonce issued by the MN, then, the MN verifies (*N*_1_ + 1), which means an attacker is authenticated to the MN. When an attacker is verified, the MN completes the rest of authentication.

### Anonymity

The SPAM method does not preserve the MN anonymity. An attacker can easily find the *ID*_*MN*_ using the intercepted login request and smart card secrets. Firstly, an attacker extracts *E*_*C*_4__(*AUTH*_*MN*_ ∥ *N*_1_) in the login request message, (*AID*_*MN*_, *C*_3_, *E*_*C*_4__(*AUTH*_*MN*_ ∥ *N*_1_)), and decrypts it using *C*_4_ to get *N*_1_. After obtaining *N*_1_, the *ID*_*MN*_ can be retrieved by computing, *ID*_*MN*_ = *AID*_*MN*_ ⊕ *h*(*C*_5_ ∥ *N*_1_), because an attacker received (*AID*_*MN*_) from login request, and (*C*_5_) from smart card. Secondly, *ID*_*MAG*_ can be retrieved from the message, (*ID*_*MAG*_, *E*_*C*_4__((*N*_1_ + 1)∥*N*_2_ ∥ *h*(*ID*_*MAG*_ ∥ *N*_2_))), as this message is sent by the MAG to the MN in a plain text, during the mutual authentication phase. Clearly, the anonymity of user is not protected because an attacker can find the ID of network entity.

### Lack of Revocation of Smart Card

The revocation procedure is used in case of the MN misbehavior or lost mobile device. The user can report the loss of the mobile device to the AAA server to prevent the further security problems like impersonation attack [30] in case of the lost or stolen mobile device. The revocation procedure is not provided for the SPAM method.

### Password Guessing Attack

In this section, we show that how an attacker can retrieve the MN password using intercepted login message based on the reference [49, 50]. An attacker can get the value, (*AID*_*MN*_, *C*_3_, *E*_*C*_4__(*AUTH*_*MN*_ ∥ *N*_1_)) and the stored information inside the smart card, (*ID*_*MN*_, *C*_1_, *C*_2_, *C*_3_, *C*_4_, *C*_5_, *h*()). From the equation, *C*_2_ = *h*(*PW*_*MN*_) ⊕ *C*_1_, as an attacker knows *C*_1_ and *C*_2_, he can compute *h*(*PW*_*MN*_) = *C*_1_ ⊕ *C*_2_. Now, he can guess a password $PW_{MN}^{*}$ and compute $h(PW_{MN}^{*})$, then check if $h\left(PW_{MN}^{*}\right)=h\left(PW_{MN}\right)$, if so, then an attacker possesses *PW*_*MN*_.

## Proposed Method

In the section, our proposed enhancement is described. First, we change registration phase in the way that if even an attacker finds the secrets inside the smart card, he cannot launch impersonation attack. Subsequently, mutual authentication procedure between the MN and the MAG is proposed. The main is idea is that smart card needs user name and password of the MN to calculate other secrets and initiate authentication.

### Initial Registration Procedure

**Fig 5:**
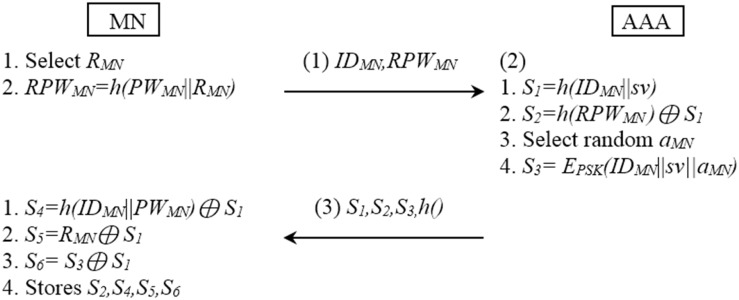
Registration procedure of the proposed method.

In this phase, the AAA server generates the secrets for the MN. The main objective of the improvement is to prevent revealing smart card information in the case of a stolen or loss device. All the stored information in smart card should be useless for an attacker. We introduce an extra value, *R*_*MN*_, in this step. Fig 5 depicts the initial registration procedure.

### Authentication Procedure

The MN should perform mutual authentication with the MAG when it joins to the localized mobility domain. We assume that an attacker can retrieve the secrets inside the smart card if the case of the stolen or lost mobile device. The main idea of our approach is not to store critical secrets inside the smart card. The mobile user enters his ID and password to the smart card to start the authentication procedure. The proposed authentication procedure is as follows:

1. The user inserts a smart card and enters its ID and password. First, it computes *S*_1_ = *h*(*ID*_*MN*_ ∥ *PW*_*MN*_) ⊕ *S*_4_. The smart card checks if, *h*(*PW*_*MN*_) ⊕ *S*_2_ = *S*_1_, then generates *N*_1_ and computes *S*_3_ = *S*_6_ ⊕ *S*_1_, *AID*_*MN*_ = *S*_1_ ⊕ *S*_6_, and *AUTH*_*MN*_ = *h*(*S*_1_ ∥ *N*_1_).
2. *MN* → *MAG*: The authentication request is formatted as *AID*_*MN*_, *E*_*S*_1__(*AUTH*_*MN*_ ∥*N*_1_) and sent to the MAG by the MN.
3. The MN verification by the MAG: After receiving the authentication request, the MAG decrypts *AID* = *S*_1_ ⊕ *S*_6_ = *E*_*PSK*_(*ID*_*MN*_ ∥ *sv* ∥ *aMN*) to obtain *ID*_*MN*_, *aMN* and *sv* using *PSK*, which is a pre-shared symmetric key between the MAG and AAA. Then, it computes *S*_1_ = *h*(*ID*_*MN*_ ∥ *sv*) to decrypt *E*_*S*_1__(*AUTHM*_*NM*_ ∥ *N*_1_) and retrieve *AUTH*_*MN*_ and *N*_1_. To obtain the *ID*_*MN*_, the MAG computes *C*_5_ and gets *ID*_*MN*_ = *AID*_*MN*_ ⊕ *h*(*C*_5_ ∥ *N*_1_). After computing *S*_1_ = *h*(*ID*_*MN*_ ∥ *sv*), the MAG can verify the MAG authentication by checking the value of *AUTH*_*MN*_ = *h*(*S*_1_ ∥ *N*_1_) to the value of *AUTH*_*MN*_ obtained from *E*_*S*_1__(*AUTH*_*MN*_ ∥ *N*_1_). If both *AUTH*_*MN*_ values are the same, the MN is authenticated and the MAG generates *N*_2_, *SK*_*MN* − *MAG*_ = *h*(*N*_1_ ∥ *N*_2_) that is a session key between the MAG and the MN, and *h*(*ID*_*MAG*_ ∥ *N*_2_).
4. *MAG* → *MN*: The MAG replies *E*_*S*_1__((*N*_1_ + 1)∥*N*_2_ ∥ *ID*_*MAG*_ ∥ *h*(*N*_2_ ∥ *ID*_*MAG*_) back to the MN.
5. The MAG verification: The MN decrypts *E*_*S*_1__(*N*1 + 1)∥*N*_2_ ∥ *h*(*N*_2_ ∥ *ID*_*M*_ *AG*)) to obtain (*N*_1_ + 1) and *N*_2_. Then, it checks the value of *h*(*N*_2_ ∥ *ID*_*MAG*_) and (*N*_1_ + 1) for the MAG authentication. After verifying the MAG authenticity, the MN generates a session key, *SK*_*MN* − *MAG*_ = *h*(*N*_1_ ∥ *N*_2_).
6. *MN* → *MAG*: The MAG computes *E*_*SK*_*MN* − *MAG*__(*N*_2_ + 1), and sends it to the MAG.
7. The MAG decrypts the received message using the session key and checks (*N*_2_ + 1) to prevent replay attack.

**Fig 6:**
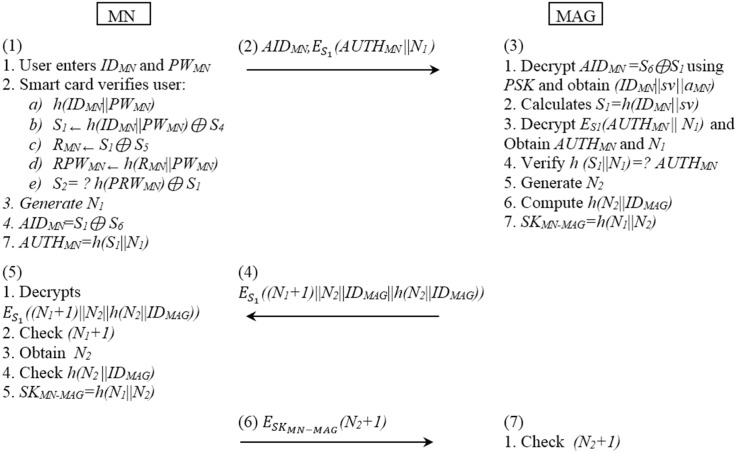
The Proposed authentication procedure between the MN and the MAG.

This mutual authentication between the MN and the MAG is described in Fig 6.

### Password Change Phase

**Fig 7:**
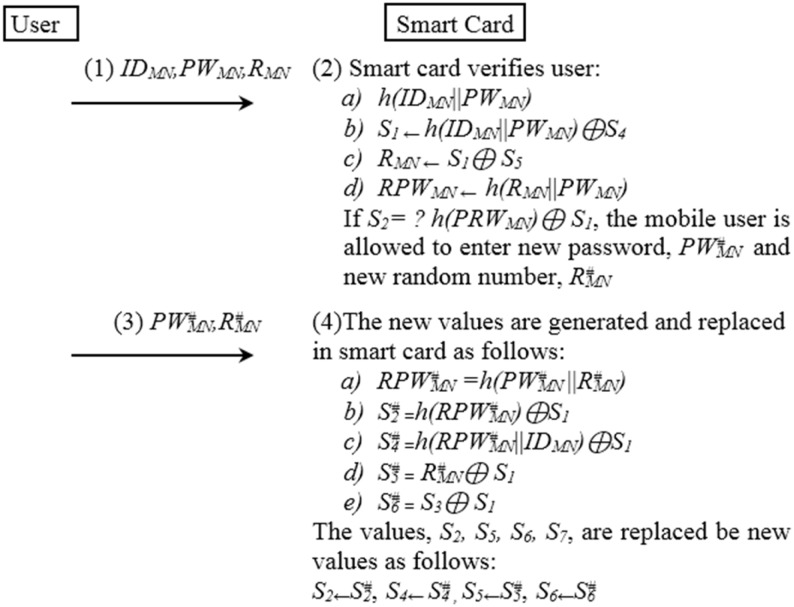
The password change procedure of the proposed method.

We improved the password change phase as described in Fig 7. It is worth noticing that the random number, *R*_*MN*_, should be changed as well the user password, *PW*_*MN*_. The symbol,♯, means the new value in Fig 7.

It worth noticing the mutual authentication procedure between the MAG and the LMA in our proposed method is the same as the SPAM method.

### Revocation Procedure

The revocation phase can be applied for the SPAM authentication scheme to protect the network entities in case of lost or stolen of smart card. Firstly, the mobile user requests the AAA server for its revocation. Then, the AAA server checks the user credentials, which can be the values known by the user. In case of revocation, the AAA server revokes all the secrets of the mobile user and creates a new set of secrets for the mobile user. Later on, the mobile user can re-register to the AAA server.

## Security Analysis of the Proposed Scheme

In this section, we analyze the security and privacy of the proposed enhanced method. Furthermore, the security comparison of the SPAM authentication scheme is provided to prove the security improvement of our proposed method. The proposed authentication method satisfies following requirements:

### Anonymity

We applied two methods to protect the MN and the MAG anonymity. For the MN anonymity, we generate an alias ID for the MN, *AID*_*MN*_ = *E*_*PSK*_(*ID*_*MN*_ ∥ *sv* ∥ *aMN*). The ID of the mobile node is mixed with *aMN*, and secret key *sv*. An adversary cannot find *ID*_*MN*_ the without knowing the secret key *PSK*. Furthermore, the use of *aMN* and *sv* restricts the adversary to launch identity guessing attack. Furthermore, in the SPAM scheme, the *ID*_*MAG*_ is transferred in the plain text during mutual authentication between the MN and the MAG. In our proposed methods; we mix the *ID*_*MAG*_ with the MAG nonce, *N*_2_, then we encrypt using one-way hash function and *N*_2_ in the message, *E*_*S*_1__((*N*_1_ + 1)∥*N*_2_ ∥ *h*(*N*_2_ ∥ *ID*_*MAG*_)). An attacker must know *N*_2_ and *N*_1_ to find the *ID*_*MAG*_, which is impossible for him because he does not know *N*_2_ and *N*_1_ even if he accesses to the smart card.

### Mutual Authentication

The mutual authentication between the MN and the MAG is provided in proposed method. As it is shown in Fig 6, the MAG checks the MN authentication in Step 3, by comparing the value, *AUTHMN* received from the MN and the value, *h*(*S*_1_ ∥ *N*_1_), where it calculates *S*_1_ = *h*(*ID*_*MN*_ ∥ *sv*). Furthermore, the MN checks the MAG authenticity is Step 5 by checking the value of *h*(*N*_2_ ∥ *ID*_*MAG*_) and (*N*_1_ + 1). Actually, the mobile node checks the value of its nonce, *N*_1_ to be sure that the MAG is legitimate, as the authentic MAG has the pre-shared secrets to decrypt the received messages from the MN.

### Revocation Procedure

The revocation of the lost mobile device is provided in proposed method to prevent further security threats against the PMIPv6. In case of loss or stealing the mobile device, the mobile user can inform the AAA server and request to revoke his secret credentials. Therefore, the mobile user can re-register to the AAA server.

### Resistance to the MN Impersonation Attack

An attacker must know some values such as *S*_1_, *S*_6_, *ID*_*MN*_, and *N*_1_ to generate the required values, *AID*_*MN*_ = *E*_*PSK*_(*ID*_*MN*_ ∥ *sv* ∥ *aMN*) and *AUTH*_*MN*_ = *h*(*S*_1_ ∥ *N*_1_) and impersonate the MN. Under the assumption of not using tamper-proof smart card; we assume that an attacker can accesses to the smart card, *S*_2_, *S*_4_, *S*_5_, *S*_6_, and even sniffs the communication messages, he cannot find out the values, *AID*_*MN*_, and *AUTH*_*MN*_ because he does not know the values, *S*_1_, *S*_3_, *ID*_*MN*_, and *R*_*MN*_.

### Resistance to the MAG Impersonation Attack

To impersonate the MAG, an attacker must know the value, *S*_5_, which is the symmetric key between the network entities, to decrypt the sniffed message, *E*_*S*_1__((*N*_1_ + 1)∥*N*_2_ ∥ *h*(*N*_2_ ∥ *ID*_*MAG*_)). Furthermore, both the MN and the MAG nonce are required to decrypt this message.

### Resistance to Replay Attack

A nonce is used for both the MN and the Mag during authentication procedure to prevent replay attack in the proposed method. Therefore, if an attacker intercepts the authentication communication messages and accesses to the secrets inside the smart card, he cannot replay the sniffed messages, as the MAG or the MN rejects the request because of using invalid nonce by an attacker.

### Forgery Attack Resistance

In this section, we discuss that a valid MN cannot launch forgery attack. If an attacker uses the it secrets, *S*_2_, *S*_4_, *S*_5_, *S*_6_, to forge another valid MN, it is impossible to find *AUTH*_*MN*_ because he does not know the AAA secret key, *sv*, to calculate *S*_1_ = *h*(*ID*_*MN*_ ∥ *sv*), an then use it to get *AUTH*_*MN*_ = *h*(*S*_1_ ∥ *N*_1_). As explained in Fig 6, the valid MN must calculate *AUTH*_*MN*_ to initiate authentication procedure.

### Denial-of-service Attack Resistance

The denial-of-service (DoS) can be discussed in two different situations in our proposed method. First, when the mobile user inserts wrong username and password during the login phase, if there is no suitable mechanism, the smart card processes some procedure and sends the login request to the MAG. In our proposed method, the smart card checks the username and password of the mobile user before computing login request. As described in Fig 6, Step 1, the smart card checks the validity of the mobile user before generating *N*_1_ and the rest of procedure. Second, an attacker can launch DoS attack by requesting password change; however, the smart card first checks *PW*_*MN*_ and *R*_*MN*_ before updating with new values, $PW_{MN}^{♯}$ and $R_{MN}^{♯}$. Therefore, DoS cannot happen by requesting password change message.

### Resistance to Password Guessing Attack

In the proposed method, an attacker should know at least *ID*_*MN*_, to find *RPW*_*MN*_ for guessing the password, which is impossible as we protect the mobile user privacy by using alias ID of the MN, *AID*_*MN*_ instead of real mobile node ID, *ID*_*MN*_. Furthermore, even an attacker can get to find *ID*_*MN*_; he cannot guess the password because he does not know the *R*_*MN*_ to calculate *RPW*_*MN*_ = *h*(*PW*_*MN*_ ∥ *R*_*MN*_).

### Stolen-verified Attack Resistance

The verification table is not required for the AAA server in our method. Therefore, an attacker cannot obtain the authentication secrets of the MN, even if he can access to the AAA server data base. In addition, the MAG does not need the verification table to verify the mobile node authenticity. In other words, even if the MAG reveals the MN secrets, an attacker cannot find another required information for authentication procedure. The security and privacy comparison between SPAM scheme and the proposed enhancement is summarized in Table 2.

**Table 2:** Comparison between proposed scheme and Chuang *et al*.s scheme.

Security Feature	SPAM	Proposed scheme
Anonymity	No	Yes
Mutual authentication	Yes	Yes
Revocation procedure	No	Yes
Resistance to the MN impersonation attack	No	Yes
Resistance to the MAG impersonation attack	No	Yes
Resistance to replay attack	Yes	Yes
Forgery attack resistance	Yes	Yes
Denial-of service attack resistance	Yes	Yes
Resistance to password guessing attack	No	Yes
Stolen-verified attack resistance	No	Yes

## Formal Security Analysis

Formal security analysis techniques are commonly used to analyze and evaluate various authentication schemes. According to literature [51–59], many security analysis methods can be employed to evaluate authentication methods. These methods can be categorized into three groups [60]; modal logic such as BAN logic [61], and GNY [62]; theorem proving; model checking such as AVISPA [63] and ProVerif [64]. In this paper, we used both security theorems and BAN logic.

### BAN Logic

BAN logic is widely used to analyze security vulnerabilities of security schemes. It consists of three main steps, including translating a target scheme into an idealized version, defining assumption, and applying BAN logic rules to achieve the intended beliefs. The notations of this logic are described in Table 3.

**Table 3:** BAN logic notations.

*P*⊲*X*	P see X
*P*∣≡*X*	P believes X
*P*∣⇒*X*	P has jurisdiction over X
*P*∣∼*X*	P once said X
#(*X*)	X is fresh
$P\overset{K}{↔}Q$	P and Q may use a shared key, K
(*X*)_*K*_	X is encrypted using, key, K

In order to evaluate the security scheme, BAN logic rules should be applied. We just use some of these rules as follows:

R1: Message-meaning rule: $\frac{P|\equiv P\overset{K}{↔}Q,P⊴{\left(X\right)}_{K}}{P|\equiv Q|∼X}$
R2: Jurisdiction rule: $\frac{P|\equiv Q|\Rightarrow X,P|\equiv Q\equiv X}{P|\equiv X}$
R3: Freshness-conjuncatenation rule: $\frac{P|\equiv \#(X),P|\equiv Q∼X}{P|\equiv Q|\equiv X}$
R4: Break conjuncatenation rule: $\frac{P|\equiv (X,Y)}{P|\equiv X,P|\equiv Y}$

The main goals of our proposed method are mutual authentication between the MN and the MAG. Furthermore, both the MN and the MAG should believe in the shared key. Based on BAN logic and our objectives, the goals of our proposed method are as follows:

- $\text{Goal}\;1:MAG|\equiv MN|\equiv (MN\overset{h(ID_{MN}∥sv)}{↔}MAG)$
- $\text{Goal}\;2:MAG|\equiv (MN\overset{h(ID_{MN}∥sv)}{↔}MAG)$
- $\text{Goal}\;3:MN|\equiv MAG|\equiv (MN\overset{h(ID_{MN}∥sv)}{↔}MAG)$
- $\text{Goal}\;4:MN|\equiv (MN\overset{h(ID_{MN}∥sv)}{↔}MAG)$

After identifying the main objectives of our proposed method, the communication messages are transformed to the idealized version.

$M1.1:MN\to MAG:(MAG\overset{PSK}{↔}AAA,MN\overset{h(ID_{MN}∥sv)}{↔}AAA,ID_{MN},sv,aMN,PSK)K_{PSK}$
$M1.2:MN\to MAG:(MN\overset{h(ID_{MN}∥sv}{↔}AAA,MN\overset{h(ID_{MN}∥sv)}{↔}MAG,N_{1},ID_{MN},h({ID}_{MN}∥sv))h({ID}_{MN}∥sv)$
$M2:MAG\to MN:(MN\overset{h(ID_{MN}∥sv}{↔}AAA,MN\overset{h(ID_{MN}∥sv)}{↔}MAG,N_{1},N_{2},{ID}_{MAG},h({ID}_{MN}∥sv))h({ID}_{MN}∥sv)$
$M3:MN\to MAG:\left(MN\overset{h(ID_{MN}∥sv}{↔}MAG,N_{2},h\left(ID_{MN}∥sv\right)\right)h\left({ID}_{MN}∥sv\right)$

The initial assumptions of our proposed method are as follows:

1. A1:MAG∣≡♯aMN
2. $A2:MAG|\equiv ♯N_{MAG}^{x}$
3. $A3:MN|\equiv ♯N_{MN}^{x}$
4. $A4:MN|\equiv (MN\overset{h(ID_{MN}∥sv}{↔}AAA)$
5. $A5:MAG|\equiv (MAG\overset{PSK}{↔}AAA)$
6. $A6:MN|\equiv MAG\Rightarrow (MN\overset{h(ID_{MN}∥sv}{↔}MAG)$
7. $A7:MAG|\equiv MN\Rightarrow (MN\overset{h(ID_{MN}∥sv}{↔}MAG)$

In this section, we analyzed our proposed method based on idealized messages and the assumptions using BAN logic rules. The proofs are as follows:

1. According to message M1.1 and assumptions A5 (message-meaning rule):
**S1**: *MAG*∣≡*MN*∣∼
$\left(MAG\overset{PSK}{↔}AAA,MN\overset{h(ID_{MN}∥sv)}{↔}AAA,ID_{MN},sv,aMN,PSK\right)$
2. According to S1 and assumptions A1 (freshness-conjuncatenation):
**S2**: *MAG*∣≡*MN*∣≡
$\left(MAG\overset{PSK}{↔}AAA,MN\overset{h(ID_{MN}∥sv)}{↔}AAA,ID_{MN},sv,aMN,PSK\right)$
3. According to message S2 and BAN logic break conjuncatenation rule:
$S3:MAG|\equiv MN|\equiv (MN\overset{h(ID_{MN}∥sv)}{↔}AAA)$
4. According to message M1.2 and S3 (message-meaning rule):
**S4**: *MAG*∣≡*MN*∣∼
$(MN\overset{h(ID_{MN}∥sv)}{↔}AAA,MN\overset{h(ID_{MN}∥sv)}{↔}MAG,N_{1},ID_{MN},h\left(ID_{MN}∥sv\right))$
5. According to S4 and assumptions A1 (freshness-conjuncatenation):
**S5**: *MAG*∣≡*MN*∣≡
$\left(MN\overset{h(ID_{MN}∥sv)}{↔}AAA,MN\overset{h(ID_{MN}∥sv)}{↔}MAG,N_{1},ID_{MN},h\left(ID_{MN}∥sv\right)\right)$
6. According to message S5 and BAN logic break conjuncatenation rule:
$S6:MAG|\equiv MN|\equiv (MN\overset{h(ID_{MN}∥sv)}{↔}MAG)$ **(Goal 1)**
7. According to message S6 and A7 and BAN logic jurisdiction rule:
$S7:MAG|\equiv (MN\overset{h(ID_{MN}∥sv)}{↔}MAG)$ **(Goal 2)**
8. According to message M2 and assumptions A4 (message-meaning rule):
**S8**: *MN*∣≡*MAG*∣∼
$\left(MN\overset{h(ID_{MN}∥sv)}{↔}AAA,MN\overset{h(ID_{MN}∥sv)}{↔}MAG,N_{1},N_{2},ID_{MN},h\left(ID_{MN}∥sv\right)\right)$
9. According to S8 and assumptions A3 (freshness-conjuncatenation):
**S9**: *MN*∣≡*MAG*∣≡
$\left(MN\overset{h(ID_{MN}∥sv)}{↔}AAA,MN\overset{h(ID_{MN}∥sv)}{↔}MAG,N_{1},N_{2},ID_{MN},h\left(ID_{MN}∥sv\right)\right)$
10. According to message S9 and BAN logic break conjuncatenation rule:
$S10:MN|\equiv MAG|\equiv (MN\overset{h(ID_{MN}∥sv)}{↔}MAG)$ **(Goal 3)**
11. According to message S10 and A6 and BAN logic jurisdiction rule:
$S11:MN|\equiv (MN\overset{h(ID_{MN}∥sv)}{↔}MAG)$ **(Goal 4)**

## Performance Analysis

The performance of our proposed method is analyzed in this section. We evaluate authentication procedure for our proposed method and compare to SPAM (Ming-Chin *et al*., 2013). The notations used in this evaluation are provided as follows:

- *T*_*hash*_: Hash function execution time
- *T*_*xor*_: XOR operation execution time
- *T*_*sym*_: Symmetric cryptography execution time
- *T*_*ran*_: Time for generating a random number

The performance of our proposed method is evaluated according to the methodology used in [65–69] and described in Table 4. The computation time for one-way hash function, symmetric cryptography, and random number generation time [70], are 0.0005 s, 0.0087 s, and 0.063075 s respectively. The computation time for XOR operation can be ignored because it trivial compare to other operations. It worth noticing that the computation time for each cryptographic operation is calculated relatively and is not the exact amount, because computation time varies based on the computation resource of network entities. In memory efficiency section, we assume that the length of ID, PW, random number, and output of hash function, is 128 bits. Table 3 summarizes performance evaluation of our proposed method and SPAM method based on criteria such as communication cost, memory requirement, and computational cost. The proposed method requires 640 bits memory space in smart card, but SPAM requires memory storage, 768 bits. Likewise, the communication cost of the proposed scheme is 896 bits, and SPAM requires 1152 bits. Similarly, the proposed scheme also having less computation cost as compared with Chuang et al.’s scheme.

**Table 4:** Performance comparison between proposed scheme and SPAM.

Criterion	Chuang *et al*.’s scheme	Proposed scheme
SC’s memory (in bit)	6 × 128 = 768 bit	5 × 128 = 640 bit
Communication cost	9 × 128 = 1152 bit	7 × 128 = 896 bit
**Computational cost**		
Authentication (MN)	5Thash+2Txor+3Tsym+1Tran	4Thash+3Txor+3Tsym+1Tran
Authentication (MAG)	5Thash+1Txor+4Tsym+1Tran	3Thash+0Txor+4Tsym+1Tran
Total	10Thash+3Txor+7Tsym+2Tran 0.20015 S	7Thash+3Txor+7Tsym+2Tran

## Conclusion

In this paper, we show that how an attacker can launch different attacks such as impersonation attack and password guessing attack using smart card secrets and sniffed login request message on Chuang et al.’s scheme. Furthermore, other security flaws such as lack of revocation procedure in case of loss or stolen device, and anonymity issues of this scheme, are discussed. In addition, we proposed an enhanced scheme to cover the discussed security drawbacks. The security of the proposed scheme is analyzed using BAN logic. The results show that proposed scheme while mitigating all the discussed security flaws, is also more efficient in terms of memory communication and computation costs.
