# Correction: Cryptanalysis and Improvement of "A Secure Password Authentication Mechanism for Seamless Handover in Proxy Mobile IPv6 Networks"

**DOI:** 10.1371/journal.pone.0145975

**Published:** 2015-12-23

**Authors:** Mojtaba Alizadeh, Mazdak Zamani, Sabariah Baharun, Azizah Abdul Manaf, Kouichi Sakurai, Hiroaki Anada, Hassan Keshavarz, Shehzad Ashraf Chaudhry, Muhammad Khurram Khan

The sixth author’s name is spelled incorrectly. The correct name is Hiroaki Anada.
